# Information Perspective to Probabilistic Modeling: Boltzmann Machines versus Born Machines

**DOI:** 10.3390/e20080583

**Published:** 2018-08-07

**Authors:** Song Cheng, Jing Chen, Lei Wang

**Affiliations:** 1 Institute of Physics, Chinese Academy of Sciences, Beijing 100190, China; 2 School of Physical Sciences, University of Chinese Academy of Sciences, Beijing 100049, China; 3 Center for Computational Quantum Physics, Flatiron Institute, New York, NY 10010, USA

**Keywords:** born machine, tensor network, mutual information

## Abstract

We compare and contrast the statistical physics and quantum physics inspired approaches for unsupervised generative modeling of classical data. The two approaches represent probabilities of observed data using energy-based models and quantum states, respectively. Classical and quantum information patterns of the target datasets therefore provide principled guidelines for structural design and learning in these two approaches. Taking the Restricted Boltzmann Machines (RBM) as an example, we analyze the information theoretical bounds of the two approaches. We also estimate the classical mutual information of the standard MNIST datasets and the quantum Rényi entropy of corresponding Matrix Product States (MPS) representations. Both information measures are much smaller compared to their theoretical upper bound and exhibit similar patterns, which imply a common inductive bias of low information complexity. By comparing the performance of RBM with various architectures on the standard MNIST datasets, we found that the RBM with local sparse connection exhibit high learning efficiency, which supports the application of tensor network states in machine learning problems.

## 1. Introduction

**Figure 1:**
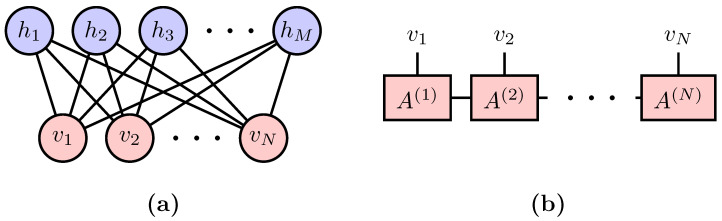
(**a**) Restricted Boltzmann Machines as a realization of the Boltzmann Machine Equation in Equation (1); and (**b**) Matrix Product States as a realization of the Born Machine Equation in Equation (2).

The fruitful interplay between statistical physics and machine learning dates back to at least the early studies of spin glasses and neural networks [1,2]. The two fields share common interests on the emergent collective behavior of complex systems with a large number of degrees of freedom. In particular, unsupervised generative modeling is closely related to the inverse statistical problems [3], where one infers the parameters of a model based on observations. The model can generate new samples according to the learned probability distribution, hence the name generative modeling. Inspired by the statistical physics, one can model the data probability according to the Boltzmann distribution with an energy function of the observed variables

(1) $$p\left(v\right)=\frac{e^{-E(v)}}{Z},$$

where $Z=\sum_{v}e^{-E(v)}$, the partition function, is the normalization factor of the probability density. The functional form of E(v), which is usually called the energy function in statistical physics, is typically predetermined to deliver certain prior knowledge about the data. Structured probabilistic models of the form in Equation (1) are collectively denoted as energy-based models [4], in which the prominent examples are the *Boltzmann Machine* [5]. Figure 1a shows an example of the Restricted Boltzmann Machines (RBM) [6] whose energy function reads $E_{\mathrm{RBM}}\left(v\right)=-\sum_{i}a_{i}v_{i}-\sum_{j}\ln\left(1+e^{b_{j}+\sum_{i}v_{i}W_{ij}}\right)$ [7]. The second term is the result of tracing out hidden units *h* which couples to the visible units v via the coupling matrix *W*.

On the other hand, by exploiting the inherent probabilistic nature of quantum mechanics, one can model the probability distribution of classical data using a quantum pure state

(2) $$p\left(v\right)=\frac{{\left|\Psi \left(v\right)\right|}^{2}}{N},$$

where $N=\sum_{v}{\left|\Psi \left(v\right)\right|}^{2}$ is the normalization factor. The square ensures the positivity of the probability. Recently, in conjunction with the applications of machine learning techniques to quantum physics problems [8,9,10,11,12,13,14,15,16], there emerges a quantum perspective to problems in machine learning [17,18,19,20,21,22,23,24,25]. In particular, Equation (2) translates the generative modeling of probability density to the problem of learning a quantum state. In fact, the necessity of this quantum interpretation was also anticipated in earlier machine learning literature. The mathematical structure of quantum mechanics appears naturally when one explores more flexible models than Equation (1) while still attempts to ensure the positivity of the probability density [26,27]. We call these approaches *Born Machines* to acknowledge the probabilistic interpretation of the quantum mechanics [28]. Besides preparing the quantum state using an actual quantum devise [29], one can employ many of the efficient classical ansatz developed by quantum physicists to represent the quantum many-body state. An example is the matrix product states (MPS) shown in Figure 1b, which is one of the simplest tensor network states. The MPS parameterizes the wavefuntion amplitude of a state of *N* variables as $\Psi_{\mathrm{MPS}}\left(v\right)=\mathrm{Tr}\prod_{i=1}^{N}A^{\left(i\right)}\left[v_{i}\right]$, where $A^{\left(i\right)}$ is a matrix for an given vector $v_{i}$.

To illustrate the contrast between Boltzman Machines and Born Machines, you can imagine that Boltzman Machines maps the probability distribution of the images into a very complex potential function. The sample with high probability corresponds to the local minimum of the potential function. The process of the image generating is similar to statistical mechanics hopping between different local minimums. In Born Machines, the probability distribution of images is mapped to a pure quantum state in Hilbert space. Each basis of this Hilbert space corresponds to one of all possible image patterns. The projection of the pure state on each basis corresponds to the probability of each image pattern. Therefore, the process of the image generating is more like the collapse of a quantum state.

Both Equations (1) and (2) allow one to import insights and concepts of statistical and quantum physics, such as symmetry, locality, sparseness and entanglement [30], to unsupervised generative modeling. Physical considerations can be used to assess the complexity, such as entropy or mutual information [31,32], of the dataset and the representational power of the corresponding models [9,10,18]. Moreover, one can employ the mathematical and computational tools developed for statistical and quantum physics for machine learning. For example, mean-field theory and Markov chain Monte Carlo methods originate from statistical physics research are by now standard tools for learning structured probabilistic models [33]. Furthermore, we anticipate that approaches in quantum physics such as tensor networks and quantum algorithms will play an increasingly significant role in generative modeling through the quantum inspired representation of probabilities (Equation (2)).

The purpose of this paper is to compare and contrast the Boltzmann Machines (Equation (1)) and Born Machines (Equation (2)) approaches for probabilistic modeling, therefore build up a unified view and motivate future studies, especially for the quantum machine learning algorithm [24] and the potential of applying the tensor network method into the machine learning problems [17,20,21]. Classical and quantum information theories provide crucial guidelines for such comparison. Classical information theory lays a common foundation for many problems in machine learning and statistical physics [34,35]. On the other hand, quantum information theory has played a crucial role in characterizing, modeling and simulating quantum states of matter [36]. For example, many methods with polynomial parameters can successfully model quantum states with exponential possibilities. It turns out the reason is many of the physically interesting quantum states only occupy a tiny corner of the Hilbert space, which fulfills the area law of the entanglement entropy [37]. Similar observations were independently made in the machine learning community [4,30] that the natural images encountered in machine learning applications occupy a negligible proportion of the volume of all possible images. This should result in the sparseness of the classic information of the images. If this is true, then modeling the probability distribution of classical dataset in terms of the quantum states would become reasonable (Equation (2)), insights for modeling quantum states [36,37] can be transferred into generative modeling of classical data.

Although early works have noticed this similarity, further research on them has not progressed much, because calculating the information of high-dimensional data such as pictures is very challenging and still a cutting-edge research area. In this work, we try to deepen the similarity between the classical Boltzmann model and the quantum Born model from the information perspective. In the theoretical analysis aspect, we point out the formalism similarity of classical and quantum information, which could brings the same statistical bias. Furthermore, we prove an inequality to make the theoretical analysis of information more useful for model design. In the numerical experiments, we applied Annealed Importance Sampling, MPS and RBM with only local sparse connection to further verify that the information in dataset of natural images is indeed local and sparse.

The organization of this paper is as follows. Section 2 defines the complexity of a dataset from the classical and quantum information theoretical perspectives. Section 3 discusses the implication of the information theoretic considerations on the probabilistic modeling using the restricted Boltzmann machines. These two sections together point out the similarities in the formulas of the information quantity of those two models and proved the inequalities between them. Section 4 compares the information measures in natural images with the information upper bond, which further illustrate from the perspective of natural images data that the two models have similar characterizations. Then, we carried out numerical experiments on the standard MNIST dataset to support our claims that the information in natural images data is sparse and local. Finally, Section 5 summarizes our main points and outlook for future directions.

## 2. Complexity of Dataset: Classical Mutual Information and Quantum Entanglement Entropy

Modeling data probability using an energy based model (Equation (1)) calls for a classical information theoretical analysis. Mutual information (MI) is a fundamental information theoretical concept which quantifies the complexity of probability distribution π(v) associated with the dataset. Assuming x∈X and y∈Y are two subset of the variables and v=x∪y, their marginal probability distributions are $\pi \left(x\right)=\sum_{y\in Y}\pi \left(x,y\right)$, and $\pi \left(y\right)=\sum_{x\in X}\pi \left(x,y\right)$, respectively. The MI reads

(3) $$I\left(X:Y\right)=\sum_{x\in X,y\in Y}\pi \left(x,y\right)\ln\left[\frac{\pi (x,y)}{\pi (x)\pi (y)}\right],$$

The MI measures the amount of information shared between the two sets of variables. MI is zero only for independent variables. In this sense, the MI is a stronger criterion than the correlation of variables since having zero correlation does not necessarily imply vanishing MI. The MI can be used as the objective functions in machine learning applications [38,39,40]. Here, we adopt a different point view, which treats MI as a complexity measure of the dataset to be modeled.

On the other hand, if we view the target dataset as snapshots of the same quantum state collapsed on a fixed basis (Equation (2)), it is natural to measure its complexity using the second Rényi entanglement entropy

(4) $$S^{R}=-\ln\mathrm{Tr}\left(\rho_{X}^{2}\right),$$

where ${\left(\rho_{X}\right)}_{x,x^{\prime }}=\sum_{y\in Y}\Psi \left(x,y\right)\Psi \left(x^{\prime },y\right)$ is the reduced density matrix, and Ψ(v=x∪y) is the probability amplitude associated with the probability, such that p(v) in Equation (2) approaches to the data probability distribution π(v). The second Rényi entanglement entropy is a lower bound of the von Neumann entanglement entropy $S^{\mathrm{vN}}=-\mathrm{Tr}\left[\rho_{X}\ln\left(\rho_{X}\right)\right]$.

To reveal connection of the classical and quantum information theoretical measures, we write the MI as

(5) $$I\left(X:Y\right)=-{\langle \ln{\langle \frac{\pi \left(x,y^{\prime }\right)\pi \left(x^{\prime },y\right)}{\pi \left(x^{\prime },y^{\prime }\right)\pi (x,y)}\rangle }_{x^{\prime },y^{\prime }}\rangle }_{x,y},$$

and the second Rényi entropy as

(6) $$S^{R}=-\ln{\langle {\langle \frac{\Psi \left(x,y^{\prime }\right)\Psi \left(x^{\prime },y\right)}{\Psi \left(x^{\prime },y^{\prime }\right)\Psi (x,y)}\rangle }_{x^{\prime },y^{\prime }}\rangle }_{x,y},$$

where the expected value ${\langle \cdots \rangle }_{x,y}$ is with respect to the dataset probability π(x,y).

**Figure 2:**
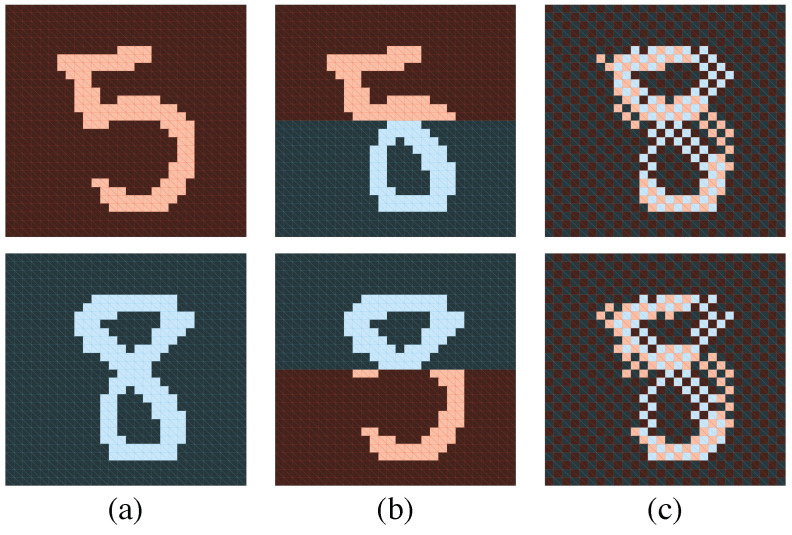
Illustration of the swap operation in Equations (5) and (6) using handwritten digits from the MNIST dataset: (**a**) two original images; (**b**) swapped images for up/down bipartition; and (**c**) swapped images for checkerboard bipartition of the pixels. The blue and red colors indicate the regions of the bipartition X and Y, respectively.

There are apparent similarities between Equations (5) and (6). Both equations contain swap ratios of probability or probability amplitude [41,42]. To illustrated the effect of the swap ratio, Figure 2a shows two samples from the MNIST dataset ((x,y) and $\left(x^{\prime },y^{\prime }\right)$) and Figure 2b,c shows the corresponding swapped images ($\left(x^{\prime },y\right)$ and $\left(x,y^{\prime }\right)$) for up/down and checkerboard bipartitions. The ratio in Equations (5) and (6) would be smaller if the swapped images are less likely to appear in the original dataset π(v), and therefore make larger contribution to the mutual information or the entanglement entropy. Earlier work argued that the dominant correlations in the natural datasets encountered in physics and machine learning applications are the local ones due to the physical law of the nature [43]. Therefore, it is natural to expect that the checkerboard bipartition (Figure 2c) has higher MI and entanglement entropy compared to the up/down bipartition (Figure 2b) because of strong local correlations between nearby pixels of natural images. Similar discussions on the information measures of different bipartitions were also considered in machine learning [19] and in quantum physics [44,45] studies.

The formal similarity between Equations (5) and (6) underlines the analogy between modeling classical data and modeling quantum states [17,18,19,20,21,22,23,24]. Quantum entanglement entropy is not merely a “metaphorical vehicle” to measure the complexity of classical dataset, but is also of practical relevance if one models the data using the quantum approach (Equation (2)). Since the general theories about the entanglement entropy scaling for various quantum states [37] are very instructive for estimating required resources to model the target quantum states, developing similar theory for typical datasets in machine learning would be very helpful for selecting generative models.

There are nevertheless differences in the two information measures of Equations (5) and (6). First, the swap operation in Equation (5) is defined for the probability density other than the quantum wavefunction. The probability amplitude may contain phase information which is however irrelevant to probabilistic modeling of the dataset [20]. Second, the logarithmic functions is sandwiched between two expectations in Equation (5), which hiders direct Monte Carlo estimate of the MI similar to the Rényi entanglement entropy [41,42]. To circumvent this difficulty, one may consider computing alternative quantities such as the Rényi mutual information [46].

## 3. Probabilistic Modeling Using Restricted Boltzmann Machine 

As a concrete example, we consider the RBM [47] for probabilistic modeling. RBM is a prominent approach for generative modeling with deep connections to statistical physics. It has also played an important role in the recent resurgence of deep learning [48,49]. Recently, the RBMs have attracted heated attentions in the quantum many-body physics community. Viewed as a variational ansatz for quantum states [8,50], the representational power of RBM was investigated from a quantum entanglement [9] and computational complexity theory [10] perspectives. Moreover, its connection to the tensor network states was explored extensively [11,14,15,16,18]. Besides representing quantum states, RBMs also find applications in identifying order parameters, quantum error correction and accelerating Monte Carlo simulations [51,52,53,54,55,56]. The later applications adopted the conventional usage of the RBMs, i.e., modeling probability density of observed data.

Conventionally, the RBM models probability distribution of data via an energy-based model with hidden units. By tracing out the hidden variables, the RBM represents a probability distribution of the visible variables. RBM can in principle approximate any probability density by using a sufficiently large number of hidden units [7,57,58,59,60,61]. However, one should note that these theorems mostly concern about the worst cases and do not take into account of typical distributions of interests. It is thus crucial to exploit the inductive bias of the RBM in terms of the information measures and match them to the characteristics of the target dataset. To do this, we define the mutual information $I_{\mathrm{RBM}}$ and entanglement entropy $S_{\mathrm{RBM}}^{R(\mathrm{vN})}$ of the RBM analogously to Equations (3) and (4), except that we now use the probability density p(v) and the corresponding probability amplitude of the RBM.

**Figure 3:**
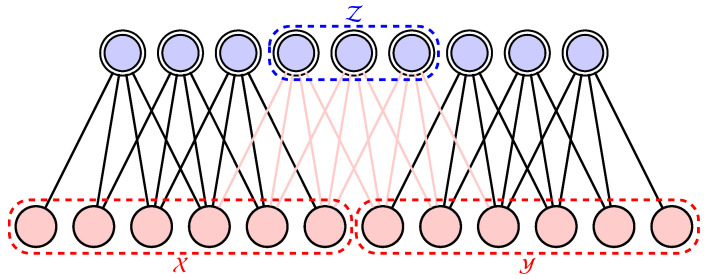
A restricted Boltzmann machine consists of visible neurons (red) and hidden neurons (blue with double line) coupled together. The two sets of visible variables X and Y are independent once the hidden variables in Z are given. The red lines are the connections that mediate the interactions between X and Y via Z.

Given an RBM architecture, one can identify two set of visible variables X,Y are connected via a minimal set of hidden variables Z (see Figure 3). The variables X and Y are independent once all the values of Z are given. This conditional independence property is denoted symbolically as X⊥Y|Z in the probabilistic graphical model notation [33]. The MI between the regions X and Y can be captured by the RBM is bounded by the size of the intermediate region

(7) $$\begin{array}{c} I_{\mathrm{RBM}}\left(X:Y\right)\leq I_{\mathrm{RBM}}\left(X:Z\right)\leq \left|Z\right|\ln2, \end{array}$$

where |Z| denotes the number of hidden units in the set Z. The factor ln2 is due to the binarization of the data in this paper. The first inequality follows directly from the data-processing inequality [62], which states that the information cannot be increased through a random channel. Alternatively, one can show that $I_{\mathrm{RBM}}\left(X:Y\right)\leq I_{\mathrm{RBM}}\left(X:Y\cup Z\right)$ using the strong subadditivity property of the MI [63] and note that $I_{\mathrm{RBM}}\left(X:Y\cup Z\right)=I_{\mathrm{RBM}}\left(X:Z\right)$ [64]. The second inequality in Equation (7) uses the fact that mutual information is bounded by the size of the subsystem. We note that the mutual information of target data is used for structural learning of fully visible probabilistic graphical model of tree structures [65]. For RBMs, information theoretical studies have mostly focused on the MI between the visible and hidden variables [66,67,68]. According to Equation (7), one can arrange the hidden neurons of an RBM into a deep architecture, thus to enlarge the size of the intermediate region and increase the expressibility of the information measures. This motivates the deep Boltzmann Machines [69] for more challenging classical datasets with even larger mutual information.

On the other hand, one can repurpose the RBM to represent the quantum state [8], i.e., the probability amplitude shown in Equation (2). In terms of the entanglement entropy, the representational power of RBM is also limited by its connectivity [9,10,11,18],

(8) $$S_{\mathrm{RBM}}^{R}\leq S_{\mathrm{RBM}}^{\mathrm{vN}}\leq \left|Z\right|\ln2,$$

Equations (7) and (8) quantify the expressibility of the RBM in terms of information theoretical measures solely by its architecture. For an RBM with dense connection, the region Z will span to all the hidden units irrespective of the information pattern of the target dataset. Information perspective provides a guiding principle for RBM architecture design conditioned on the typical information pattern of the target dataset. Equation (7) shows that two sets of visible variables of an RBM should connect to at least I(X:Y))/ln2 hidden neurons to adequately capture the MI of the dataset. We anticipate that the MI of natural images and physical model should be much smaller than the maximum value $\min\left(|X|,|Y|\right)\ln2$ due to physical nature of the probability distributions. The connectivity of the RBM puts a constraint on the maximum information that can be captured, therefore limits its expressibility. Conversely, this also provides an inductive bias towards natural datasets of low information complexity. Interpreting the generative modeling in terms of capturing the MI or entanglement of the target dataset sheds new light on the learning process.

An important question relevant to quantum machine learning is to identify realistic datasets which are significantly easier to model in the quantum approach than the classical approach [70]. In light of the above discussion, one is inclined to look for those cases in datasets where the entanglement entropy lower bound in Equation (8) is much smaller than the classical mutual information lower bound in Equation (7). We verified numerically that in general there is no definite inequality between Equations (5) and (6). Therefore, it would be interesting to construct explicit examples where the quantum approach requires fewer resources.

One should nevertheless be careful when drawing the analogy between modeling quantum states and classical datasets. For example, it is sometimes argued that one needs deep neural nets to model classical dataset with critical correlations in analog to critical quantum systems which can only be captured by hierarchical tensor networks [71]. However, the scaling behavior of the mutual information of a critical classical system is different from the entanglement entropy of a critical quantum system. The MI of statistical physics model with short-range interactions scales only with the boundary size between subsystems [64], which holds irrespective whether the system is critical or not. As a concrete example, the critical Ising model only requires a shallow RBM to be modeled exactly [18], which is in line with the area law scaling of its mutual information [72,73]. Other results [55] also showed that deep architectures do not seem to exhibit advantages for modeling the critical Ising data.

We also mensioned the work [12] using an RBM to model the probability of a quantum state on a fixed basis for quantum state tomography. Since the approach corresponds to Equation (1), the required resources are determined by the Shannon mutual information of quantum states [74,75], instead of the entanglement entropy. The two entropies exhibit similar scaling behavior for the examples discussed in Refs. [74,75]. However, in general, this may not be the case. Thus, it remains open to see whether it is advantageous to use an RBM to model the probability or using a complex valued RBM to model the quantum state directly.

## 4. Information Pattern of MNIST Dataset and Its Implication to Generative Modeling

**Figure 4:**
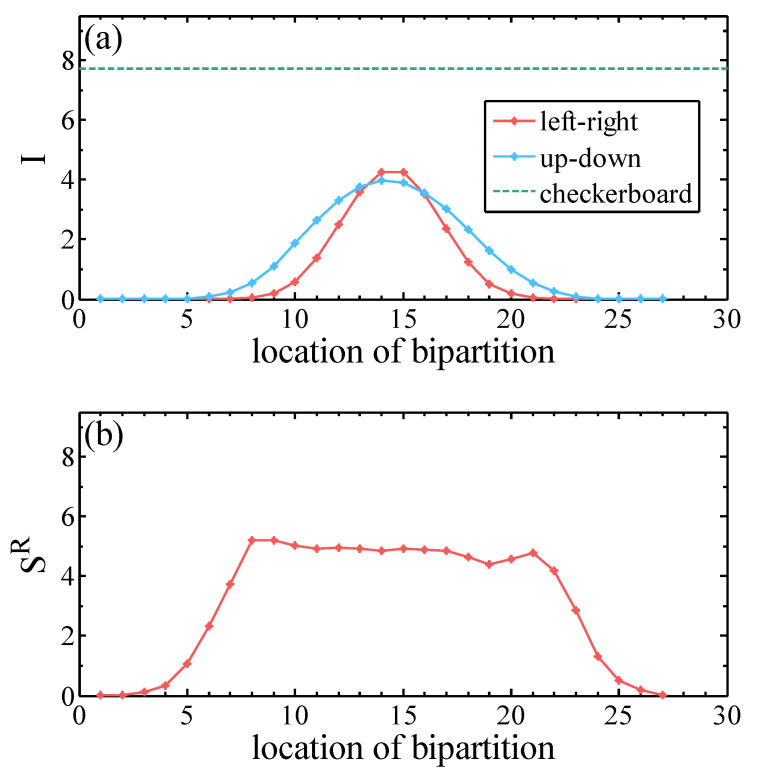
(**a**) Classical mutual information (Equation (3)) of 10,000 MNIST dataset for various bipartitions of images; and (**b**) Rényi entropy (Equation (4)) of 10,000 MNIST dataset by well-trained MPS.

We considered generative modeling the MNIST dataset by exploiting the information pattern of the target dataset. First, it is extremely challenging to accurately compute the MI of high-dimensional distribution such as the images with 784 pixels. We employed the approach of Ref. [76] to estimate the MI of the MNIST dataset. The approach is based on the nearest neighbor estimate of the Shannon entropy widely adopted in the statistics literature. Figure 4a shows how the MI increases as one cuts into the center of the image. The vertical and horizontal bipartition of the images exhibits a similar behavior of MI. The MI between the margin and the remaining part of the image is zero since the margin of the MNIST image is always fixed.

The maximum of MI is much smaller than its theoretical limit in Equation (7), which indicates densely connected RBM has significant redundancy. Meanwhile, MI of the checkerboard bipartitions (e.g., Figure 2c) is significantly higher than the left/right or up/down bipartitions. This suggests that introducing hidden units which couple to the nearby pixels are more efficient in capturing the MI of the MNIST dataset.

One should note that the MI estimator [76] is only approximate, especially for highly dependent variables [77]. It is generally a difficult task to estimate the MI of image dataset rigorously. On the other hand, estimating the Reńyi entropy (6) is feasible by using tensor network [17,20,21] or Monte Carlo approaches [41,42]. We estimated the Reńyi entropy of the MNIST dataset in Figure 4b. First, we employed the approach of [20] to train an MPS on 10,000 MNIST images. When the MPS is trained well, we calculate its reduced density matrix ${\left(\rho_{X}\right)}_{x,x^{\prime }}=\sum_{y\in Y}\Psi \left(x,y\right)\Psi \left(x^{\prime },y\right)$ at each bipartition position. The goodness of the learning is measured by the negative log-likelihood (NLL) evaluated on the dataset $L=-\frac{1}{\left|D\right|}\sum_{v\in D}\ln\left[\frac{{\left|\Psi \left(v\right)\right|}^{2}}{N}\right]$ where |D|=10000. In this case, the NLL of MPS for training data is 39.634. Then, we obtained the second Reńyi entropy (Equation (4)). The second Reńyi entropy can be used as a measure of the correlation between two parts of the pictures by left-right bipartition or up-down bipartition, depending on the lay out of the MPS on the 2D image. The value of Reńyi entropy in the MNIST dataset has reached the one of the 2D quantum spin system computed using DMRG [78], which shows a similar level of complexity. Interestingly, not only its value, but also the distribution of the Reńyi entropy exhibit similar behavior as the mutual information, which confirms the connection between Equations (5) and (6).

Both the maximum value of the mutual information in RBM and the maximum value of the Reńyi entropy in MPS are much smaller than their theoretical maxima. They both suggest that MI are highly redundancy. The traditional fully-connected models of equal weights have great redundancy and are very inefficient under the condition of same number of parameters. This can be easily checked by numerical experiment. For example, we can compare the performance of a fully connected model with a sparsely connected model under same number of parameters.

For sparsely connected model, the relatively higher values of the checkboard bipartition than the up-down/left-right bipartition suggests the local connections are more important for capturing the dataset probability distribution. If this is correct, we could expect a sparsely connected model with only sparse local connection can perform relatively well than the sparse random connected model. This assumption also can be easily checked by numerical experiments.

We confirm these two assumptions by training RBMs with the same number of parameters but with different connection architectures and different number of hidden neurons. Dense connection means that the visible and hidden units of the RBM are fully connected and hidden units will be less than visible units. For 1D, 2D and Random RBM, they have the same number of hidden and visible neurons. Random means that we randomly connect the visible and hidden neurons. While 1D connection means that each hidden neurons of the RBM is connected only to a $2l_{1}+1$ fragment of the entire image vector, where $l_{1}$ is denoted as the 1D connection length, see Figure 3, 2D RBM means that each hidden neuron connects to a small $2\left(l_{2}+1\right)\times \left(l_{2}+1\right)$ window of a 2D image, where $l_{2}$ is denoted 2D connection length.

The goodness of the learning is measured by the NLL evaluated on the test dataset $L=-\frac{1}{\left|D\right|}\sum_{v\in D}\ln\left[\frac{e^{-E(v)}}{Z}\right]$, where |D| is the size of the test set. To compute the NLL one has to estimate the intractable partition function, for which we employed the Annealed Importance Sampling approach [79,80]. The estimated NLL provides an up bound of the entropy of the dataset, which also bounds the MI between two arbitrary division of the variables, i.e., L≥I(X:Y).

**Figure 5:**
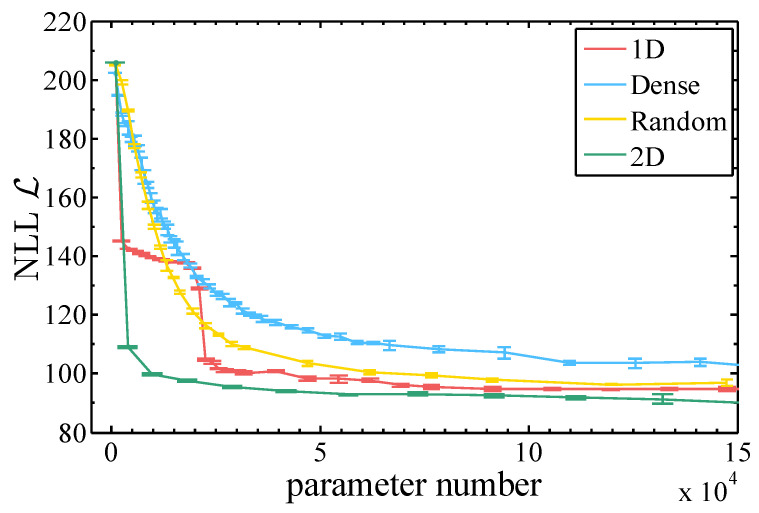
Negative log-likelihood for various RBM architectures plotted against the number of parameters in the model.

One clearly sees that, in Figure 5, the RBM structure with local connections which respects the 2D nature of the images reaches the lowest NLL quickly, and with the least number of parameters, while the NLL of the RBM with 1D connections exhibits an abrupt drop when the two nearby pixels from the different row are connected. The RBM with dense connections performs even worse than the random connections with the same number of parameters.

**Figure 6:**
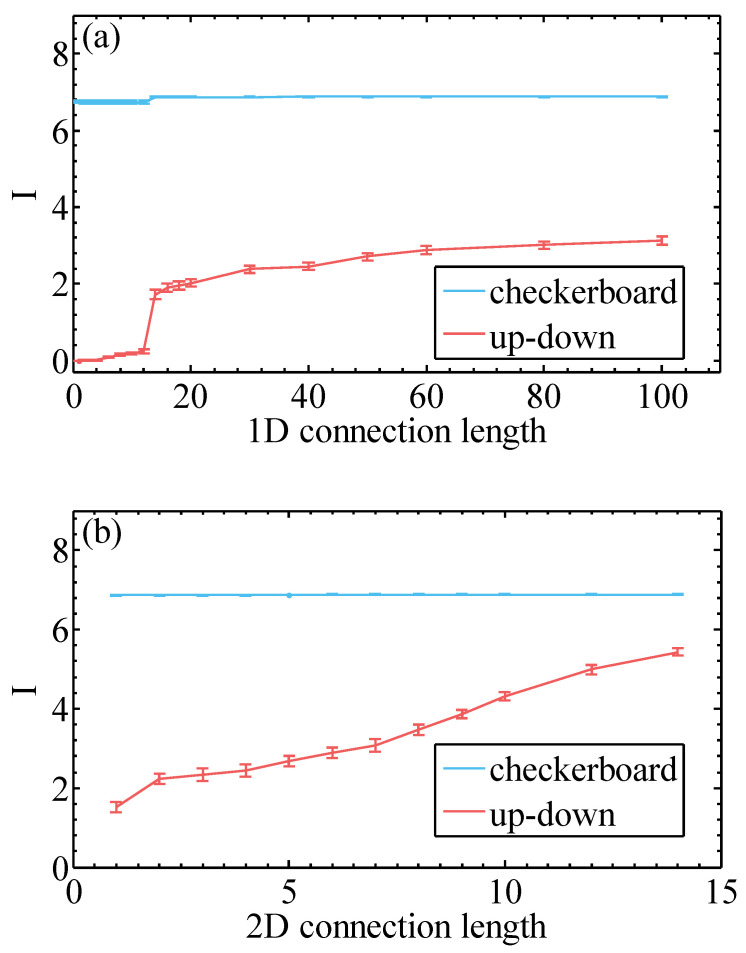
(**a**) Mutual information Equation (5) of 10,000 MNIST dataset for various bipartitions of images. The result is estimated with trained 1D sparse local RBM. (**b**) Mutual information of 10,000 MNIST dataset for various bipartitions of images. The result is estimated with trained 2D sparse local RBM.

To further reveal the connection between the captured MI and the quality of the learned RBM, we calculated the MI of different 1D and 2D sparse RBMs (Figure 6) via Equation (5). The MI of 1D sparse RBMs exhibits an abrupt increase at $l_{1}=14$, which is just the moment when the fragment is long enough to be able to include two neighboring pixels from different row. Compared with Figure 5, the abrupt increase of MI corresponds exactly to the sudden drop of NLL. Meanwhile, 2D sparse RBMs captures more MI than the RBM with 1D connections. From these comparisons, we see that learning via minimizing the NLL is also a process of learning MI of the dataset.

Our results on the NLL of sparse RBM, and the surprisingly small MI and Renyi entropy, are consistent with the previous experiments on RBMs with sparse connections [81,82,83,84]. Earlier work [85] showed the neural network works fine, even with 80% randomly dropped out. Moreover, A sparsely connected RBM with small-world network structure and found that it performs well compared with a densely connected RBM is also been proposed [86]. Since RBMs with local and sparse connections has close connections to the tensor networks which can be handled in practical computation [18], these results support the applications of tensor network states in practical machine learning problems [17,20,21]. In those applications, it is natural to adopt Equation (2) and the associated quantum information perspectives in machine learning problems.

## 5. Summary

In summary, revealing the similarity of the two information theoretical measures Equations (5) and (6) suggests that the statistical physics and quantum physics inspired approaches for generative modeling using Boltzmann Machines (Equation (1) and Born Machines (Equation (2)) have similar inductive biases. Therefore, successful wavefunction representations in quantum physics have the potential to be good generative models for machine learning, and vice versa.

Classical and quantum information theories shed light on the expressibility and architecture design of generative models. Our discussions and numerical experiments suggest that it is rewarding to design architectures which take into information pattern of the target dataset. In particular, imposing locality greatly increases the learning efficiency by exploiting the mutual information structure of the typical dataset. This is akin to the success of the convolutional neural network structure for discriminative tasks.

Besides the expressibility issue discussed in this paper, learning and sampling of the energy-based models can be slow due to the intractable partition functions. Conventionally, this is solved by using Markov chain Monte Carlo sampling or mean-field theory approaches. The quantum representation offers an alternative solution to these problems. For example, modeling the probability amplitude as matrix product states or tree tensor networks offer advantages in efficient learning and sampling [17,20,21]. Moreover, representing the probability distribution using a quantum state [24,87] obviously permits efficient sample generation by simply performing measurement to the quantum state.

In the above discussions, we have separated the energy-based models and quantum state representations for probabilistic modeling of classical data. Nevertheless, a combined approach with a “quantum statistical model” is also possible, in which one models the classical probability density using mixed quantum states. In this respect, the quantum Boltzmann machines [88,89,90] can be viewed as an example. Finally, this paper focuses on modeling the probability of data without labels. In a general setting, one could also model the joint probability distribution of the data and label. In this case, one can generate samples conditioned on the class label and elaborate on the entanglement entropy of each class individually [21].

## References

[1] Hopfield J.J. (1982). Neural networks and physical systems with emergent collective computational abilities. Proc. Natl. Acad. Sci. USA.

[2] Amit D.J., Gutfreund H., Sompolinsky H. (1985). Spin-glass models of neural networks. Phys. Rev. A.

[3] Nguyen H.C., Zecchina R., Berg J. (2017). Inverse statistical problems: From the inverse Ising problem to data science. Adv. Phys..

[4] Goodfellow I., Bengio Y., Courville A. (2016). Deep Learning.

[5] Hinton G.E., Sejnowski T.J. (1986). Learning and relearning in Boltzmann machines. Parallel Distributed Processing: Explorations in the Microstructure of Cognition.

[6] Hinton G.E. (2012). A Practical Guide to Training Restricted Boltzmann Machines. Neural Networks: Tricks of the Trade.

[7] Barra A., Bernacchia A., Santucci E., Contucci P. (2012). On the equivalence of hopfield networks and boltzmann machines. Neural Netw..

[8] Carleo G., Troyer M. (2017). Solving the quantum many-body problem with artificial neural networks. Science.

[9] Deng D.L., Li X., Das Sarma S. (2017). Quantum entanglement in neural network states. Phys. Rev. X.

[10] Gao X., Duan L.M. (2017). Efficient representation of quantum many-body states with deep neural networks. Nat. Commun..

[11] Huang Y., Moore J.E. (2017). Neural network representation of tensor network and chiral states. arXiv.

[12] Torlai G., Mazzola G., Carrasquilla J., Troyer M., Melko R., Carleo G. (2017). Many-body quantum state tomography with neural networks. arXiv.

[13] Cai Z. (2017). Approximating quantum many-body wave-functions using artificial neural networks. arXiv.

[14] Clark S.R. (2017). Unifying neural-network quantum states and correlator product states via tensor networks. J. Phys. A Math. Theor..

[15] Glasser I., Pancotti N., August M., Rodriguez I.D., Cirac J.I. (2017). Neural-networks quantum states, string-bond states and chiral topological states. Phys. Rev. X.

[16] Kaubruegger R., Pastori L., Budich J.C. (2017). Chiral topological phases from artificial neural networks. Phys. Rev. B.

[17] Miles Stoudenmire E., Schwab D.J. (2016). Supervised learning with quantum-inspired tensor networks. arXiv.

[18] Chen J., Cheng S., Xie H., Wang L., Xiang T. (2017). On the equivalence of restricted boltzmann machines and tensor network states. arXiv.

[19] Levine Y., Yakira D., Cohen N., Shashua A. (2017). Deep learning and quantum entanglement: fundamental connections with implications to network design. arXiv.

[20] Han Z.Y., Wang J., Fan H., Wang L., Zhang P. (2017). Unsupervised generative modeling using matrix product states. arXiv.

[21] Liu D., Ran S.J., Wittek P., Peng C., García R.B., Su G., Lewenstein M. (2017). Machine learning by two-dimensional hierarchical tensor networks: A quantum information theoretic perspective on deep architectures. arXiv.

[22] Zhang Y.H. (2017). Entanglement entropy of target functions for image classification and convolutional neural network. arXiv.

[23] Pestun V., Vlassopoulos Y. (2017). Tensor network language model. arXiv.

[24] Gao X., Zhang Z., Duan L. (2017). An efficient quantum algorithm for generative machine learning. arXiv.

[25] Huang Y. (2017). Provably efficient neural network representation for image classification. arXiv.

[26] Bailly R. Quadratic weighted automata: Spectral algorithm and likelihood maximization. Proceedings of the Asian Conference on Machine Learning.

[27] Zhao M.J., Jaeger H. (2010). Norm-observable operator models. Neural Comput..

[28] Born M. (1926). Zur Quantenmechanik der Stoßvorgänge. Z. Phys..

[29] Benedetti M., Garcia-Pintos D., Nam Y., Perdomo-Ortiz A. (2018). A generative modeling approach for benchmarking and training shallow quantum circuits. arXiv.

[30] Lin H.W., Tegmark M. (2016). Why does deep and cheap learning work so well?. arXiv.

[31] Battiti R. (1994). Using mutual information for selecting features in supervised neural net learning. IEEE Trans. Neural Netw..

[32] Koch-Janusz M., Ringel Z. (2018). Mutual information, neural networks and the renormalization group. Nat. Phys..

[33] Koller D., Friedman N. (2009). Probabilistic Graphical Models, Principles and Techniques.

[34] MacKay D.J. (2003). Information Theory, Inference and Learning Algorithms.

[35] Mezard M., Montanari A. (2009). Information, Physics, and Computation.

[36] Zeng B., Chen X., Zhou D.L., Wen X.G. (2015). Quantum information meets quantum matter–from quantum entanglement to topological phase in many-body systems. arXiv.

[37] Eisert J., Cramer M., Plenio M.B. (2010). *Colloquium*: Area laws for the entanglement entropy. Rev. Mod. Phys..

[38] Linsker R. (1988). Self-organization in a perceptual network. Computer.

[39] Bell A.J., Sejnowski T.J. (1995). An information-maximization approach to blind separation and blind deconvolution. Neural Comput..

[40] Alemi A.A., Fischer I., Dillon J.V., Murphy K. (2016). Deep variational information bottleneck. arXiv.

[41] Hastings M.B., González I., Kallin A.B., Melko R.G. (2010). Measuring renyi entanglement entropy in quantum monte carlo simulations. Phys. Rev. Lett..

[42] Zhang Y., Grover T., Vishwanath A. (2011). Entanglement Entropy of critical spin liquids. Phys. Rev. Lett..

[43] Lin H.W., Tegmark M., Rolnick D. (2017). Why does deep and cheap learning work so well?. J. Statis. Phys..

[44] Hsieh T.H., Fu L. (2014). Bulk entanglement spectrum reveals quantum criticality within a topological state. Phys. Rev. Lett..

[45] Rao W.J., Wan X., Zhang G.M. (2014). Critical-entanglement spectrum of one-dimensional symmetry-protected topological phases. Phys. Rev. B.

[46] Iaconis J., Inglis S., Kallin A.B., Melko R.G. (2013). Detecting classical phase transitions with Renyi mutual information. Phys. Rev. B.

[47] Smolensky P. (1986). Information Processing in Dynamical Systems: Foundations of Harmony Theory. Parallel Distributed Processing: Explorations in the Microstructure of Cognition.

[48] Hinton G., Salakhutdinov R. (2006). Reducing the dimensionality of data with neural networks. Science.

[49] Hinton G.E., Osindero S. (2006). A fast learning algorithm for deep belief nets. Neural Comput..

[50] Nomura Y., Darmawan A., Yamaji Y., Imada M. (2017). Restricted-boltzmann-machine learning for solving strongly correlated quantum systems. arXiv.

[51] Torlai G., Melko R.G. (2016). Learning thermodynamics with Boltzmann machines. Phys. Rev. B.

[52] Huang L., Wang L. (2017). Accelerated Monte Carlo simulations with restricted Boltzmann machines. Phys. Rev. B.

[53] Torlai G., Melko R.G. (2017). Neural decoder for topological codes. Phys. Rev. Lett..

[54] Wang L. (2017). Exploring cluster Monte Carlo updates with Boltzmann machines. Phys. Rev. E.

[55] Morningstar A., Melko R.G. (2017). Deep learning the ising model near criticality. arXiv.

[56] Rao W.J., Li Z., Zhu Q., Luo M., Wan X. (2017). Identifying product order with restricted boltzmann machines. arXiv.

[57] Freund Y., Haussler D. (1994). Unsupervised Learning of Distributions of Binary Vectors Using Two Layer Networks. Advances in Neural Information Processing Systems.

[58] Le Roux N., Bengio Y. (2008). Representational power of restricted Boltzmann machines and deep belief networks. Neural Comput..

[59] Montufar G., Ay N. (2011). Refinements of universal approximation results for deep belief networks and restricted Boltzmann machines. Neural Comput..

[60] Montúfar G., Rauh J. (2016). Hierarchical models as marginals of hierarchical models. Int. J. Approx. Reason..

[61] Montufar G.F., Rauh J., Ay N., Shawe-Taylor J., Zemel R.S., Bartlett P.L., Pereira F., Weinberger K.Q. (2011). Expressive Power and Approximation Errors of Restricted Boltzmann Machines. Advances in Neural Information Processing Systems 24.

[62] Cover T.M., Thomas J.A. (2012). Elements of Information Theory.

[63] Preskill J. (2016). Quantum Shannon theory. arXiv.

[64] Wolf M.M., Verstraete F., Hastings M.B., Cirac J.I. (2008). Area laws in quantum systems: Mutual information and correlations. Phys. Rev. Lett..

[65] Chow C., Liu C. (1968). Approximating discrete probability distributions with dependence trees. IEEE Trans. Inform. Theory.

[66] Berglund M., Raiko T., Cho K. (2015). Measuring the usefulness of hidden units in boltzmann machines with mutual information. Neural Netw..

[67] Peng K.H., Zhang H. Mutual information-based RBM neural networks. Proceedings of the 2016 23rd International Conference on Pattern Recognition (ICPR).

[68] Koch-Janusz M., Ringel Z. (2017). Mutual information, neural networks and the renormalization group. arXiv.

[69] Salakhutdinov R., Hinton G.E. Deep Boltzmann machines. Proceedings of the Twelfth International Conference on Artificial Intelligence and Statistics (AISTATS’09).

[70] Perdomo-Ortiz A., Benedetti M., Realpe-Gómez J., Biswas R. (2018). Opportunities and challenges for quantum-assisted machine learning in near-term quantum computers. Q. Sci. Technol..

[71] Vidal G. (2008). Class of quantum many-body states that can be efficiently simulated. Phys. Rev. Lett..

[72] Wilms J., Troyer M., Verstraete F. (2011). Mutual information in classical spin models. J. Statis. Mech. Theory Exp..

[73] Lau H.W., Grassberger P. (2013). Information theoretic aspects of the two-dimensional Ising model. Phys. Rev. E.

[74] Alcaraz F.C., Rajabpour M.A. (2013). Universal behavior of the Shannon mutual information of critical quantum chains. Phys. Rev. Lett..

[75] Stéphan J.M. (2014). Shannon and Rényi mutual information in quantum critical spin chains. Phys. Rev. B.

[76] Kraskov A., Stögbauer H., Grassberger P. (2004). Estimating mutual information. Phys. Rev. E.

[77] Gao S., Steeg G.V., Galstyan A. Efficient estimation of mutual information for strongly dependent variables. Proceedings of the International Conference on Artificial Intelligence and Statistics (AISTATS).

[78] Depenbrock S., McCulloch I.P., Schollwöck U. (2012). Nature of the Spin-Liquid Ground State of the *S* = 1/2 Heisenberg Model on the Kagome Lattice. Phys. Rev. Lett..

[79] Neal R.M. (2001). Annealed importance sampling. Statis. Comput..

[80] Salakhutdinov R., Murray I., McCallum A., Roweis S. On the quantitative analysis of Deep Belief Networks. Proceedings of the 25th Annual International Conference on Machine Learning (ICML 2008).

[81] Tubiana J., Monasson R. (2017). Emergence of compositional representations in restricted boltzmann machines. Phys. Rev. Lett..

[82] Agliari E., Barra A., Galluzzi A., Guerra F., Moauro F. (2012). Multitasking associative networks. Phys. Rev. Lett..

[83] Sollich P., Tantari D., Annibale A., Barra A. (2014). Extensive parallel processing on scale-free networks. Phys. Rev. Lett..

[84] Agliari E., Barra A., Galluzzi A., Guerra F., Tantari D., Tavani F. (2015). Retrieval capabilities of hierarchical networks: From dyson to hopfield. Phys. Rev. Lett..

[85] Dumoulin V., Goodfellow I.J., Courville A., Bengio Y. On the challenges of physical implementations of RBMs. Proceedings of the Twenty-Eighth AAAI Conference on Artificial Intelligence.

[86] Mocanu D.C., Mocanu E., Nguyen P.H., Gibescu M., Liotta A. (2016). A topological insight into restricted Boltzmann machines. Mach. Learn..

[87] Farhi E., Harrow A.W. (2016). Quantum supremacy through the quantum approximate optimization algorithm. arXiv.

[88] Amin M.H., Andriyash E., Rolfe J., Kulchytskyy B., Melko R. (2016). Quantum Boltzmann machine. arXiv.

[89] Kieferova M., Wiebe N. (2016). Tomography and generative data modeling via quantum Boltzmann training. arXiv.

[90] Benedetti M., Realpe-Gómez J., Biswas R., Perdomo-Ortiz A. (2016). Quantum-assisted learning of graphical models with arbitrary pairwise connectivity. arXiv.

